# Digital phenotyping by wearable-driven artificial intelligence in older adults and people with Parkinson’s disease: Protocol of the mixed method, cyclic ActiveAgeing study

**DOI:** 10.1371/journal.pone.0275747

**Published:** 2022-10-14

**Authors:** Juan C. Torrado, Bettina S. Husebo, Heather G. Allore, Ane Erdal, Stein E. Fæø, Haakon Reithe, Elise Førsund, Charalampos Tzoulis, Monica Patrascu

**Affiliations:** 1 Faculty of Medicine, Department of Global Public Health and Primary Care, Centre for Elderly and Nursing Home Medicine (SEFAS), University of Bergen, Bergen, Norway; 2 Department of Nursing Home Medicine, Municipality of Bergen, Bergen, Norway; 3 Yale School of Medicine and Yale School of Public Health, New Haven, CT, United States of America; 4 Faculty of Health Studies, Department of Nursing, VID Specialized University, Bergen, Norway; 5 Department of Neurology, Neuro-SysMed Center, Haukeland University Hospital, Bergen, Norway; 6 K.G Jebsen Center for Translational Research in Parkinson’s Disease, University of Bergen, Bergen, Norway; 7 Department of Clinical Medicine, University of Bergen, Bergen, Norway; Universita degli Studi della Campania Luigi Vanvitelli, ITALY

## Abstract

**Background:**

Active ageing is described as the process of optimizing health, empowerment, and security to enhance the quality of life in the rapidly growing population of older adults. Meanwhile, multimorbidity and neurological disorders, such as Parkinson’s disease (PD), lead to global public health and resource limitations. We introduce a novel user-centered paradigm of ageing based on wearable-driven artificial intelligence (AI) that may harness the autonomy and independence that accompany functional limitation or disability, and possibly elevate life expectancy in older adults and people with PD.

**Methods:**

ActiveAgeing is a 4-year, multicentre, mixed method, cyclic study that combines digital phenotyping via commercial devices (Empatica E4, Fitbit Sense, and Oura Ring) with traditional evaluation (clinical assessment scales, in-depth interviews, and clinical consultations) and includes four types of participants: (1) people with PD and (2) their informal caregiver; (3) healthy older adults from the Helgetun living environment in Norway, and (4) people on the Helgetun waiting list. For the first study, each group will be represented by N = 15 participants to test the data acquisition and to determine the sample size for the second study. To suggest lifestyle changes, modules for human expert-based advice, machine-generated advice, and self-generated advice from accessible data visualization will be designed. Quantitative analysis of physiological data will rely on digital signal processing (DSP) and AI techniques. The clinical assessment scales are the Unified Parkinson’s Disease Rating Scale (UPDRS), Montreal Cognitive Assessment (MoCA), Geriatric Depression Scale (GDS), Geriatric Anxiety Inventory (GAI), Apathy Evaluation Scale (AES), and the REM Sleep Behaviour Disorder Screening Questionnaire (RBDSQ). A qualitative inquiry will be carried out with individual and focus group interviews and analysed using a hermeneutic approach including narrative and thematic analysis techniques.

**Discussion:**

We hypothesise that digital phenotyping is feasible to explore the ageing process from clinical and lifestyle perspectives including older adults and people with PD. Data is used for clinical decision-making by symptom tracking, predicting symptom evolution, and discovering new outcome measures for clinical trials.

## Background

The term “active ageing” was coined by the World Health Organization (WHO) in the late 1990s and was defined formally in its policy framework of 2012 [1] to describe “the process of optimizing opportunities from health, participation and security in order to enhance quality of life as people age”. Autonomy, quality of life, and longer life expectancy were identified as the key factors that influence active ageing [2]. An increasing caregiving burden shows the need for a new paradigm of ageing that fosters increased autonomy [3]. Emergent digital means, such as wearable devices and home sensors, can arguably enable empowerment and self-determination, as proven by their extensive use in neurology [4,5].

Among the many causes of disability in older adults, neurological disorders are the leading ones worldwide [3]. Parkinson’s disease (PD) is the fastest growing neurological disorder, increasing faster than Alzheimer’s disease [3,6]. From 1990 to 2015, the prevalence of PD has more than doubled with consequential morbidity, disability and mortality [3]. The Global Burden of Disease Study [7] estimates that 6.2 million individuals currently have PD, and the number is expected to increase towards 14.2 million in 2040. About 40% of people with PD develop dementia within 10 years from diagnosis [8].

Clinically, PD is characterized by a combination of progressive motor and non-motor symptoms and signs. Typical motor symptoms include bradykinesia, resting tremor, rigidity, and postural instability. Non-motor dysfunction comprises a broad spectrum of features, including but not limited to olfactory loss, sleep disorders, autonomic dysfunction affecting the gastrointestinal and cardiovascular systems, dementia, and neuropsychiatric dysfunction, including apathy, anxiety, depression [9,10]. Many of these features can be measured, monitored, and possibly better managed through activity-based monitoring technologies [11].

The assessment of PD symptoms is normally done by a clinician who observes the patient perform a series of tasks [12]. Standardized instruments such as Unified Parkinson’s Disease Rating Scale (UPDRS, [13]), Montreal Cognitive Assessment (MoCA, [14]), or the REM Sleep Behaviour Disorder Screening Questionnaire (RBDSQ, [15]) assess disease development, cognitive decline and associated sleep disorders, respectively. Meanwhile, agreement exists that this form of data collection may have low degrees of reliability, reproducibility, and sensitivity to treatment effects [16].

Digital phenotyping is the moment-to-moment quantitative description of a person in their own environment obtained by automatically aggregated data (e.g., heart rate, movement or electrodermal activity) collected by devices (e.g., mobile devices, activity monitors or video) to measure human behaviour and function in health and disease [17]. Various studies concerned with the use of digital health in neurology show the potential of these technologies for clinical studies related to ageing and PD [5]. Passively collecting data through wearable sensors ensures compliance and lower attrition rates [18] and has the potential to increase the knowledge base and to support people with PD [19]. A systematic review of the literature by Rovini et al. (2017) identified 136 articles on wearable devices for the management of PD in different application fields, such as early diagnosis or home and long-term monitoring, concluding that this type of device is portable, light weight, unobtrusive, easy to use, inexpensive, and accurate in the measurements [20]. In clinical research, wearable use is supported by evidence for the capacity to monitor symptoms, minimize rater-bias and increase sensitivity to subclinical physiological changes [21]. In combination with AI methods to analyse big and complex data outputs, digital phenotyping holds vast potential [21–24]. However, few studies currently explore the precise description of active ageing, how neurological disease presents and develops in the individual, prediction of symptoms using AI, and technology adoption in different user groups of older adults.

Home-based continuous monitoring enables investigation of the interplay between health factors and living environment, which is recognized as a major factor in the ageing process [25–27]. Recently, new forms of senior housing are rapidly emerging, implying unmet needs. Some of the most common housing typologies are independent living, co-housing, and assisted living. Although these housing communities vary in embodiment, they share the same goal: support the health and well-being of older adults, enable them to remain community-dwelling and maintain autonomy longer. However, the quantification of the effects of these innovative living arrangements by digital phenotyping are unreported.

Our main hypothesis is that digital phenotyping is a feasible, sensitive, and specific tool to explore the ageing process from the clinical and the lifestyle perspectives in older adults with and without PD. Several secondary hypotheses are explored:

1. A smart, engaging living environment increases physical, social, and mental activities in older adults measurable with digital phenotyping.
2. Clinical diagnosis, symptom tracking, treatment-response, and symptom prediction in PD can be conducted with digital phenotyping.
3. Caregiver burden in PD can be quantified by digital phenotyping by including informal caregivers in clinical trials.

## Methods

ActiveAgeing is a 4-year, multicentre, mixed-method, open (not blinded) cyclic study aimed to develop a framework of digital phenotyping and traditional data collection intended to explore a data-driven, active living process for older adults with or without PD. The study will be conducted within the Western Norway Regional Health Authority and will include four types of participants:

1. People with PD (for the first study N = 15; estimated for the second study N = 90) will be included regardless of their age and gender given a life expectancy of more than 6 months. They will be recruited during outpatient visits at the Centre of Excellence for Treatment of Neurological Diseases (Neuro-SysMed), Haukeland University Hospital (HUS). The responsible neurologist (CT) will oversee identifying the potential participants and informing them about the DIGI.PARK (DIGItal phenotyping in people with PARKinson’s disease) study.
2. The spouses or informal caregivers (partners, friends) of people with PD are invited to participate in the second study (N = 90) if they have daily contact with the person with PD of minimum 4 hours. The exclusion criteria and the recruitment strategy are the same as for the participants with PD. People with PD and their spouses/caregivers will be represented as dyads to shed light on the mutual effect of PD progression.
3. Older adults (for the first study N = 15; for the second study N = 40) from the innovative Helgetun living environment, which is a popular housing concept in Bergen, Norway, aimed to engage a mental, social, and physical active living style. The inclusion criteria are regardless of age, sex, and initial health status given a life expectancy of more than 6 months. Exclusion criterion is a diagnosis of PD. Participants will be recruited through the user-representatives of Helgetun and researchers at the University of Bergen (UiB) through open presentations of the ActiveAgeing project, information packages (talks, flyers, newsletters, slide presentations, website) with contact information and a link to register their interest.
4. As Helgetun is a popular living environment, a waiting list of older adults from the Western region of Norway has been established. For the second study, we aim to include participants (N = 50) from this waiting list as a control group. The recruitment will be done by randomly contacting people on the waiting list and providing them with the information packages offered to the residents of Helgetun (described above).

The study involves one user representative associated with the UiB research group (RS), one user representative from Helgetun (KO), and the panel of user representatives from Neuro-SysMed for the DIGI.PARK branch. They will assess the recruitment process and ensure that the participants receive proper consent and project information material, ensuring participant interests are considered and incorporated from the beginning and throughout the project.

### Sample size

Given the exploratory nature of this study, in terms of application of emergent technologies unused for this purpose, formal sample size calculations were infeasible, as previous studies that inform the calculation were unavailable. The size of the first study was based on a feasible number of persons with PD from group 1 (described above) with an equivalent number of Helgetun residents (group 3 above). The second study used the size of the waiting list for Helgetun (group 4 above), which contained around 100 applicants, and we estimated that half of them would be willing to join, in a similar ratio to the number of Helgetun residents (group 3 above). The number of participants in the DIGI.PARK branch (group 1 above) in the second study is based on the sum of Helgetun residents and future residents (groups 3 and 4 above).

### Experimental configuration

**Fig 1:**
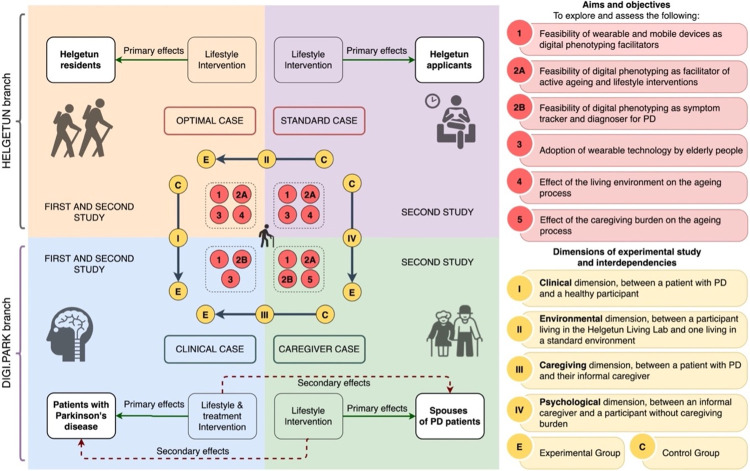
Participants in ActiveAgeing and their experimental features. I. Clinical dimension: Considering the participants with PD as the experimental group, and the participants in Helgetun as the control group, we explore the impact of a neurological condition (i.e., PD) on the ageing process. II. Environmental dimension: Considering the residents of Helgetun as the experimental group, and the people on the waiting list as the control group, we explore the impact of living in an innovative living environment like Helgetun on the ageing process. III. Caregiving dimension: Considering the participants with PD as the experimental group, and their informal caregivers as the control group, we explore the impact of a neurological condition (i.e., PD) on the ageing process in dyads of participants. IV. Psychological dimension: Considering the caregivers of people with PD as the experimental group, and the people on the waiting list for Helgetun as the control group, we explore the psychological impact of having caregiving duties on the ageing process.

Fig 1 shows the 4 categories of participants. The red discs (and their description on the upper right side) show the aims of the study, and how they are related to each participant category. The yellow discs (and their description on the lower right side) show the experimental dimensions. We envision 4 experimental configurations of participants, addressing different dimensions of the ageing process:

Fig 1 shows where the lifestyle recommendations or treatment changes that are generated from each cycle of the framework will be applied and assessed. Of note is that for the PD dyads, their respective interventions are expected to have a secondary effect on their counterpart.

### Ethics approval and consent to participate

Participants can withdraw from the study at any time, without giving any reason for doing so. Participants can withdraw their data after data collection is finished. However, data cannot be retracted after being analysed and published. All study data is recorded via digital platform (electronic form administered via tablet), transferred to and stored on a secure server in collaboration with BIOS, UiB. The project involves human data collection and processing e.g., mental, physical health, demographics, activities. Regarding *data protection* and *data privacy*, we respect the Data Protection Directive, EU Directive 95/46/EC aimed at protecting the fundamental rights and freedoms of natural persons and their right to privacy with respect to processing of personal data. We adhere to directive 2016/679, an EU-wide law including the General Data Protection Regulation (GDPR), as a significant step towards more responsible protection of individuals [28]. GDPR Article 35 requires the Data Protection Impact Assessment (DPIA), to ensure data minimization of relevant data and it limits data access to those who are authorized or given permission by the individual [28]. The project has received ethical approval to initiate the first study from the Norwegian Center for Research Data (NSD, RN-792472). Further data collections will be carried out after reviewing and renewing this ethical approval.

### ActiveAgeing framework

**Fig 2:**
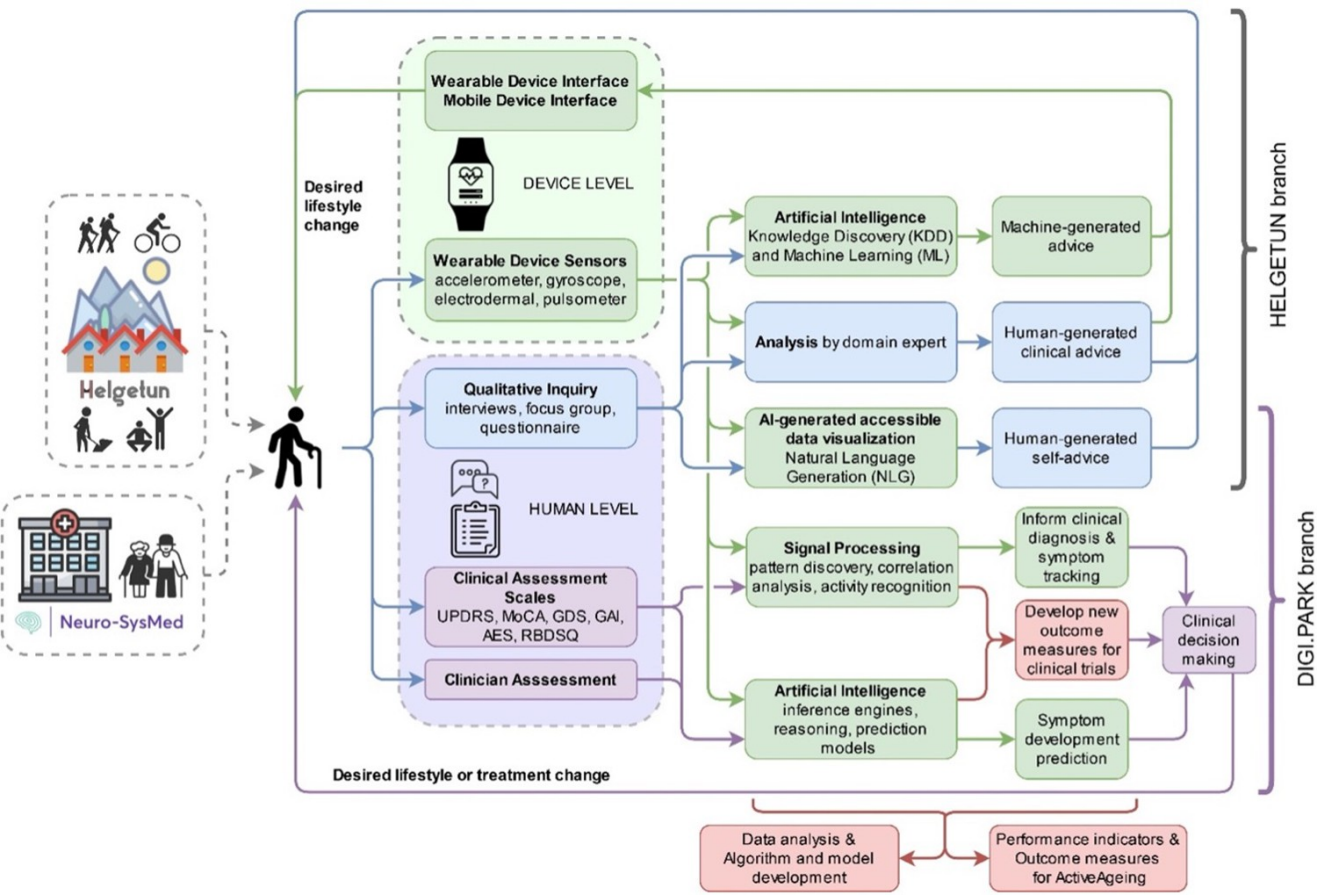
ActiveAgeing framework.

The ActiveAgeing framework describes the interaction between older adults and their data, that 1) fosters lifestyle changes towards active ageing and 2) advances the understanding of PD, in a cyclic and data-driven manner (Fig 2). The participant is at the centre of this framework, from which data is collected and upon which interventions or lifestyle changes are applied. As a generic label, “the participant” represents any participant from any category, in any iteration of the cycle, as well as caregivers and clinicians. Thus, we suggest an approach for the development of individualised lifestyle, treatment interventions, and decision making.

Additionally, this framework and the cyclic aspect of its implementation is oriented towards developing, investigating, and adapting new outcome measures to advance the understanding of PD. The impact of the living environment, the adoption of technology, and the effect of the caregiving burden on informal caregivers of people with PD are complementary targets of investigation in this study. This approach is aimed to benefit three types of stakeholders: a) older adults that will make use of the proposed ActiveAgeing framework, b) researchers investigating PD that will apply the new outcome measures in future clinical trials, and c) policy makers that will be able to use the performance indicators of the ActiveAgeing framework to allocate resources for senior care.

Two main loops can be observed in the framework: the DIGI.PARK branch and the Helgetun branch. Both loops describe different analyses made over data generated from the participants by two means.

### DIGI.PARK branch

The DIGI.PARK branch investigates PD, as a representative disorder of the neurological conditions that affect older adults. By collecting physiological data via wearable devices, data from clinical assessment scales, and clinical records of traditional consultations, we aim to open three fronts that complement each other to achieve the goal to better understand PD:

- Inform clinical diagnosis and symptom tracking: for this purpose, digital signal processing (DSP) techniques [29] such as pattern discovery, correlation analysis, and human activity recognition (HAR) [30] are applied to sensor data. Then, these findings are available to the clinician in an understandable manner, to use in diagnosis and tracking symptoms.
- Predict symptom development: PD is a complex condition, characterised by sets of interconnected symptoms varying over time and from person to person. As such, specialised models are built using inference engines and reasoning systems [31] to construct a matching set of prediction models. These models are used to estimate, within confidence intervals, the future development of one or more symptoms that can assist treatment by a clinician.
- Develop new outcome measures: clinical trials that address PD currently rely on clinical assessment scales. However, these scales carry a significant amount of subjectivity and variability (i.e., their output depends on the clinician who applies the scales) [21]. Additionally, these assessment scales provide the clinicians with rough evaluations of the symptoms that may not capture the subtleties of slighter changes that might occur because of treatment and/or treatment adjustments. Therefore, clinical trials will benefit from the objectivity and sensitivity that daily-life physiological data from digital phenotyping can provide.

### Helgetun branch

The goal of the Helgetun branch is to understand which factors foster an active lifestyle physically, mentally, and socially, in a way that empowers older adults to engage in their ageing process. Therefore, we aim to achieve this by tailoring individual advice that entails a desirable change in the behaviour and/or lifestyle of a participant. This advice is the main output of every iteration of the loop in the Helgetun branch and is delivered via a mobile phone application (developed during the ActiveAgeing development phase) that aggregates the collected physiological data. We envision three categories of advice that can be created:

- Human-generated self-advice: we apply Natural Language Generation (NLG) techniques [32,33] to create an accessible and user-friendly presentation of the data collected with the devices and the qualitative inquiry. This way, the participant will be able to understand what that data means and will have the information needed to make a lifestyle change if they deem so. This part of the framework appeals directly to our goal to empower older people to engage in their ageing process.
- Human-generated clinical advice: we rely on the domain expert to make the analysis of the collected data and tailor individual advice for the participant. A domain expert in this context would be, for instance, a nurse that reviews movement data from the sensors and suggests to the participant that they would benefit from a daily walk, or a GP that examines the patient and analyses the sleep data to suggest a treatment change. Research and dissemination of the data retrieved from the qualitative inquiry is included in this loop, which is aimed to shed light on the subjective perspectives, experiences, beliefs and reception of wearable technology and living in innovative housing environments.
- Machine-generated advice: by matching the subjective data collected at the human level to the objective data collected at the device level, we aim to discover patterns through analysis based on Knowledge Discovery from Data (KDD) [34]; then, based on these patterns, build an AI reasoning system using ML techniques (knowledge-based systems [31], context-based reasoning [35], fuzzy logic [36,37]) to generate lifestyle-changing advice that is then communicated to the user.

For both branches, we will collect data from two sources: device monitoring and human data collection (the “device level” and the “human level”, in Fig 2).

### Device level

The device level includes data from wearables generated by moment-to-moment, passive, and unobtrusive sensing of the participants’ daily lives. The following devices (2 smartwatches and 1 smart-ring) are used. All devices are commercially available. We chose one smartwatch (Fitbit Sense) with widespread popularity and higher acceptance, and another smartwatch (Empatica E4) that is oriented to research purposes and provides access to raw data with no pre-processing. The smart-ring was chosen because it represents a more innovative line of smart garments and has the potential to combine strong accuracy with high user acceptance [38].

**Fitbit Sense** [39] is a smartwatch released in 2021 under the Fitbit umbrella of devices, aiming to be the new flagship device for advanced health and activity tracking. It has a 3-axis accelerometer, gyroscope, altimeter, multipath optical heart rate tracker, ECG and EDA sensors, and on-wrist electrical sensor. It has connectivity to Android and iOS, and is waterproof.

The **Oura Ring** [40] is the first commercially available smart ring. It includes a 3-axis accelerometer, gyroscope, multipath optical heart rate tracker, and on-wrist skin temperature sensor.

The **Empatica E4** wristband [41] is a medical-grade wearable device that offers real-time physiological data acquisition, enabling researchers to conduct in-depth analysis and visualization. It is the only wearable on the market to combine electrodermal activity and heart rate sensors, simultaneously enabling the measurement of sympathetic nervous system activity and heart rate. It includes a 3-axis accelerometer, gyroscope, electrodermal activity sensor, infrared thermopile, heart rate tracker, and an event mark button.

### Software

The study uses the cloud services from Fitbit Sense (Fitbit Cloud) and Oura Ring (Oura Cloud) to initially handle the data generated by them, which follow the California Consumer Privacy Act of 2018 (CCPA) and the General Data Protection Regulation (GDPR). The software Empatica E4 Manager is used to handle its data, although it does not use a cloud service. Fitbit Studio and Jupyter Notebooks are the programming environments. Data analysis is carried out with MATLAB R2021b (using the Signal processing, Simulation, and Statistics and Machine Learning toolboxes) and SPSS version 28.0.1 for quantitative analysis, and MaxQDA 2020 for qualitative analysis.

Additionally, we use Ipsilon, an interactive task-based digital cognitive assessment tool that uses tones to evaluate brain function. It can detect early signs of dementia and mild cognitive impairment through testing attention, executive functioning, learning and memory. Ipsilon evaluates executive function, inhibition control, attention, and memory function by quantifying learning to assess brain functionalities. Specifically, Ipsilon assesses comprehension (visuo-spatial/motor encoding) and time-stamped finger responses via a web-based computerized cognitive assessment aid, running on a tablet device. Potential advantages include the elimination of language biases, relative ease and speed of operation, it is operable by a non-specialist and administered in 3–5 minutes. Ipsilon developers are currently seeking approval for status as a medical device software in the EU. DIGI.PARK plans to implement Ipsilon cognitive assessment for participants with PD in DIGI.PARK.

### Primary and secondary clinical assessment scales

People with PD are assessed with the UPDRS, which is a clinical tool for motor symptoms in PD. This data is collected by CT and HR at Neuro-SysMed. The UPDRS is comprised of four subscales: Part I, with an observer-rated assessment of six items and seven self-assessed items relating to non-motor experiences of daily living; Part II with 13 items for self-assessing the motor experiences of daily living; Part III is an 18-item observer-rated motor examination; Part IV assesses motor complications with a six-item observer-rated list, where each item is scored on a scale from 0 (normal) to 4 (severe). Scores on all items within each subscale are summarized to yield a total score for each part and discriminates between mild, moderate, and severe symptoms with cut-offs validated for each of the subscales: Part I: mild (≤10), moderate (11–21), severe (≥22); Part II: mild (≤2), moderate (13–29), severe (≥30); Part III: mild (≤32), moderate (33–58), severe (≥59); Part IV: mild (≤4), moderate (5–12), severe (≥13) [42,43]. UPDRS is among the most widely used assessment tools for motor symptoms in PD and belongs to the “gold standard” of clinical assessment tools, in which the patient performs tasks while a trained clinician visually evaluates the movements and assesses motor symptoms [44,45].

The following assessment scales are applied for all participants. The MoCA has been shown to reliably assess cognitive function in people with and without PD [46,47] and is used as a screening tool for mild cognitive impairment and dementia. The MoCA test is a 30-item tool probing various cognitive constructs including short-term memory, visuo-spatial abilities, multiple aspects of executive functioning, phonetic fluency, language, verbal abstraction, attention, concentration, subtraction, and orientation to place and time. The total score ranges from 0–30 and the cut-off for cognitive impairment is <26 [47]. The Geriatric Depression Scale (GDS) is a self-reported scale which reliably assesses depression in older people with and without PD [48,49]. The GDS is a 15-item one-dimensional scale with “yes” or “no” responses. The total score ranges from 0–15 and the cut-off for depression is ≥5. The Geriatric Anxiety Inventory (GAI) assesses anxiety and has been validated in older individuals without PD [50], as well as in people with PD without dementia [51]. The GAI is an assessment scale comprised of 20 questions with a binary “agree/disagree” response option, where a score of >10 indicates clinically significant symptoms of anxiety [50]. Starkstein Apathy Scale (SAS) consists of 14 questions with answers ranging from “not at all” to “very”. The SAS has reliably measured apathy in people with PD without cognitive impairment [52] and validated for measuring apathy in healthy older adults [53]. The RBDSQ screens for REM behaviour disorder symptoms and has been validated among people with and without PD [15,54]. The RBDSQ is a 10 item self-reported scale with binary “yes/no” responses. The questions cover the patients’ dreams, nocturnal awakenings, nocturnal vocalization, nocturnal motor movement and behaviour, and sleep-related injuries [55]. The total score ranges from 0–13 and a total score >6 indicates clinically significant symptoms [15].

All described instruments have been validated and Norwegian translations are available. A summary of the clinical assessment scales and the sensors involved in the digital phenotyping is shown in Table 1.

**Table 1:** Clinical assessment scales.

Symptom	Clinical assessment scales							Sensors				
	UPDRS	MoCA	GDS	GAI	SAS	RBDSQ	Ipsilon	IMU	HR	EDA	Temp	CSP
Tremor	X							X				
Gait	X							X				
Dyskinesia	X							X				
Bradykinesia	X											
Rigidity	X							X				
Cognition		X					X					
Depression	X		X					X	X	X	X	
Apathy					X			X	X			
Anxiety				X					X	X		
Sleep	X					X		X	X	X	X	X

**IMU:** Inertial Measurement Unit, a combination of sensors (e.g., gyroscopes, accelerometers) detecting angular momentum and acceleration; **HR:** Heart rate sensor; **EDA:** Electrodermal activity, a sensor which detects changes in skin conductance due to sweat; **Temp:** Temperature, measures body and ambient temperature; **CSP:** Colour sensitive photodiode, a light sensor.

### Qualitative methods

In-depth interviews with the participants are carried out to gather subjective data about the impact of the living environment on the ageing process, adoption of technology, and what constitutes active ageing. These interviews are framed into a hermeneutic (interpretivist and multiperspectivistic) approach aimed to capture what matters to the participants. The interviews are recorded and then transcribed and coded using MaxQDA software. This approach explores common traits and variation in how these phenomena are experienced by participants, new opportunities of interpreting and understanding these perspectives and how to apply this understanding in future interventions [56].

### Cyclic methodology overview and outcome measures

**Fig 3:**
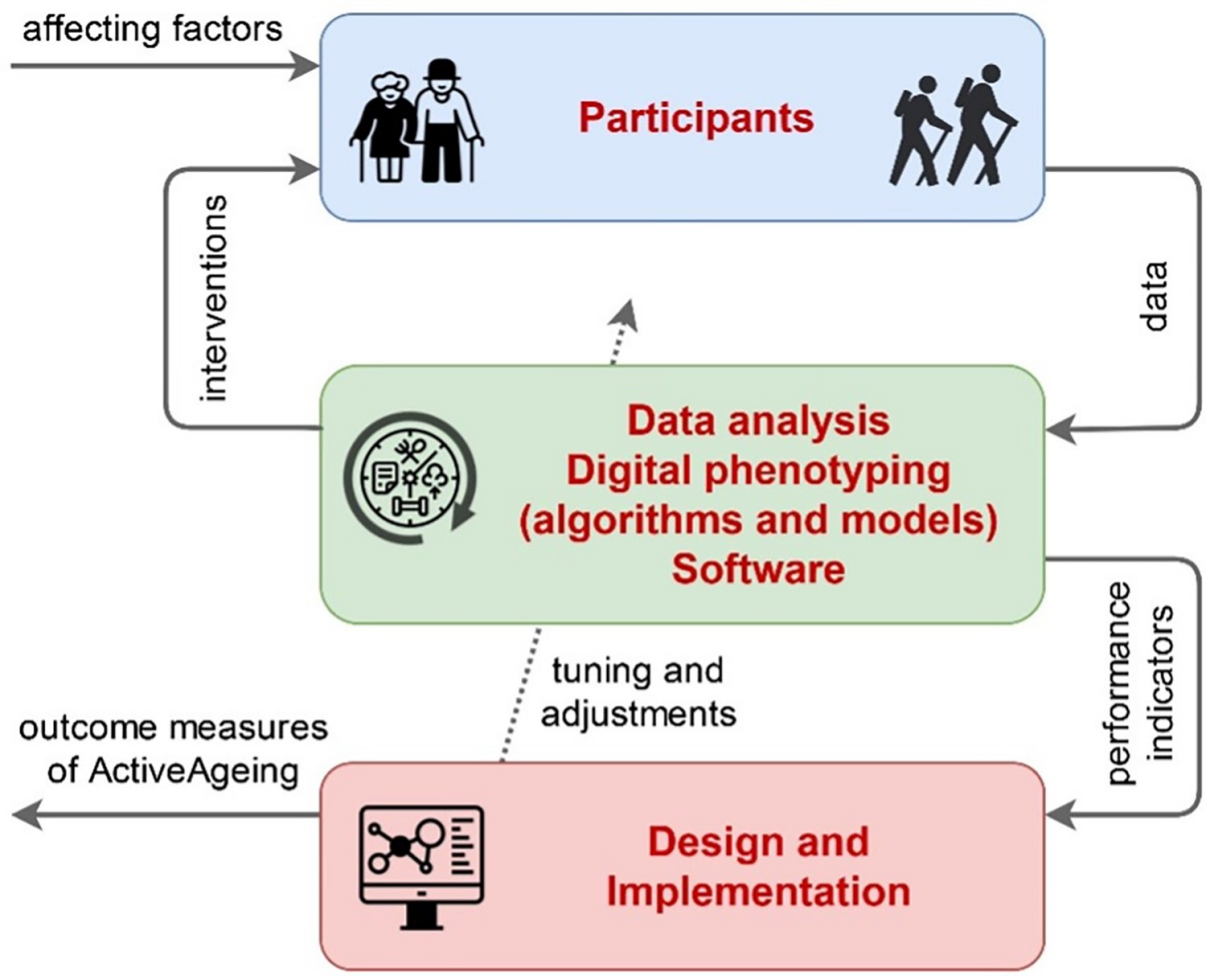
Overview of the ActiveAgeing research methodology.

**Fig 4:**
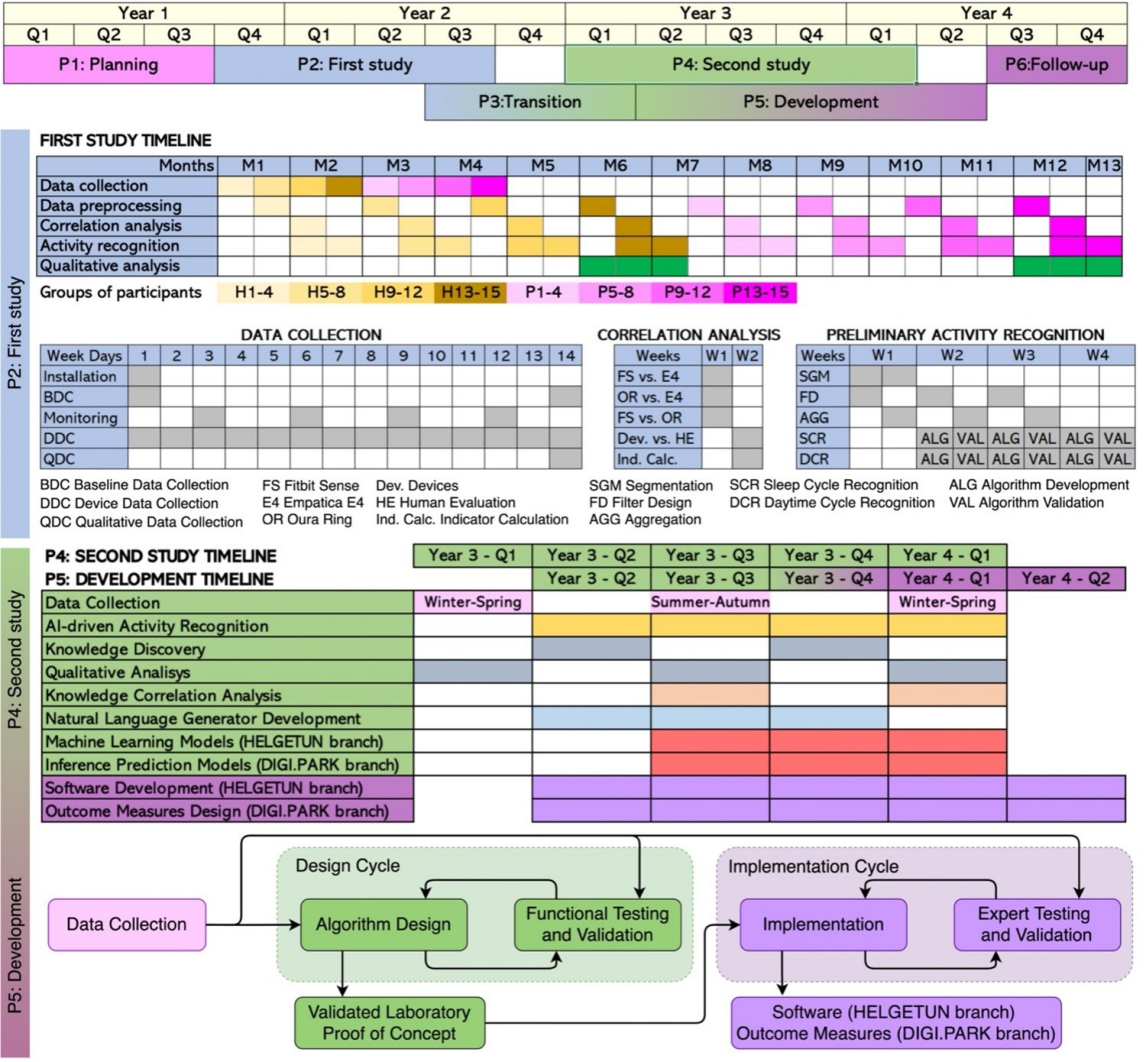
ActiveAgeing timeline and development cycle.

The ActiveAgeing framework answers the research constructs formulated in Hypotheses through a cyclic methodology (Fig 3), which is the result of the combination of AI methods and user-centred interventions. In a cyclic manner, we tune and adjust the algorithms and models that analyse the data collected from the participants, based on the performance indicators of each cycle. After the tuning process is completed, these indicators become the outcome measures of ActiveAgeing:

- Performance-based measures: standard evaluation metrics for the efficiency and accuracy of the algorithms and models are applied. For knowledge discovery and symptom classification, we use scores based on precision and recall (confusion matrix, F1 score, ROC). For dynamic models, we use standard confidence-based validation by correlating expected outputs vs. model outputs (for the same inputs), resulting in a confidence index for which a model is valid at >95–98%. Additionally, we use measures based on the error between expected and model outputs (mean, square sum, root mean square, absolute, time-weighted integral, etc.), resulting in lowest error-tolerance scores, and k-fold cross-validation. For prediction evaluation, we use accuracy (correct predictions), sensitivity (“yes” predictions), and precision (correct “yes” predictions) on each predicted variable, where the “yes/no” dichotomy is based on symptom appearance or increase/decrease (e.g., in frequency, intensity, etc.). We will evaluate the performance of these algorithms in terms of optimisation (i.e., performance analysis), given that they are meant to be executed by wearable devices, which have limited computational power and battery life. This performance analysis consists of analysing space and time complexity [57,58].
- Observer-reported measures: we will measure the effectiveness of the generated lifestyle advice by estimating the difference between the digital phenotypes obtained before and after the advice is delivered. The qualitative inquiries at every data collection round result in a set of themes that are compared against each other to analyse intra-subjective differences in perspectives and experiences around technology adoption, health and living environment. These subjective differences are treated as indicators of the feasibility and efficacy of our framework in every iteration of the cycle, and determine the way we continue using these technology, collecting data, and administering advice at future data collection points of the timeline (see Fig 4).
- Clinician reported measures: the clinical assessment scales (UPDRS, MoCA, GAI, SAS, RBDSQ) described above.

### Use case scenarios

The following use case scenarios illustrate how ActiveAgeing is to be applied to different user profiles.

**Box 1: Use case 1: Ragnhild**

Ragnhild is a female resident of Helgetun. She is 72 years old and is included in the ActiveAgeing program. She wears an Empatica E4 device on her wrist and an Oura Ring. These devices capture physiological and activity data all day. She has the ActiveAgeing app installed in her phone. Before going to bed, she checks the app, where she can look at her own data in an accessible way, which has been tailored with Natural Language Generation techniques. One night, she observes in the data that her sleep quality has decreased, and her physical activity is lower lately. Then, she decides to sign up for the winter hiking club in Helgetun. After three weeks, she observes in the app that not only her sleep quality has improved, but that she has increased her average sleeping time from 6.4 to 8.2 hours. She likes this lifestyle change and decides to continue participating in the hiking club.

At the same time, the app prompts another piece of advice to her: she might benefit from a darker bedroom, since the light sensor detected that the ambient light is bright at night.

Additionally, Ragnhild has given her GP access to her data. During her last consultation, her GP assesses her activity data in the last months. She congratulates Ragnhild for her increase of physical activity but suggests her to replace some of her hiking activities with some weekly strength training in the Helgetun gym, since she considers this type of physical activity beneficial. Then, her GP proposes to look again at her data at the next consultation to see if the change has had any observable effect.

**Box 2: Use case 2: Sigurd**

Sigurd is a 66-year-old man with PD. He was diagnosed 2 years ago by his neurologist. So far, his symptoms are tremor, balance issues, and anxiety. His right side is more heavily affected by the disease, and he has not been able to tie his own shoes lately. Trygve, his spouse, is currently performing such task for him. They sign up for the ActiveAgeing program. Sigurd wears an Empatica E4 and Oura Ring on the right (more affected) wrist and a Fitbit Sense on his left (less affected) wrist. During the last consultation, his neurologist has seen in the movement data that Sigurd’s tremor and balance issues have worsened. Consequently, his medication is adjusted, and a new appointment for 2 months later is scheduled. In the following consultation, the movement data shows that the symptoms have flattened, or they have not worsened. Sigurd is capable of tying his shoes. Next, the neurologist opens the prediction tab, where she inputs a certain dosage of his medication for the next 6 months. The algorithms run that prediction from the last 4 months data, and they infer that only a small adjustment on the dosage would be enough to alleviate the symptoms for the next 6 months.

**Box 3: Use case 3: Trygve**

Sigurd’s spouse, Trygve, is 71 years old, and is his informal caregiver. For the last two months, Trygve has had increased fatigue and depression, probably caused by Sigurd’s restless sleep, and started taking sleeping pills regularly. Then, Trygve is provided with an Empatica E4 and an Oura Ring. After Sigurd’s medication adjustment, Trygve is called by his GP to check his data. Since Sigurd sleeps better, Trygve finds it easier to sleep, and the data shows that his stress levels are lower. His GP suggests reducing his sleeping medication.

### Implementation

Fig 4 shows the implementation plan and development cycle of the ActiveAgeing framework. It consists of 6 phases across 4 years.

#### P1. Planning

The first phase lasted 9 months and includes pre-project tasks. The research group initiated contact with the user representatives and the participant groups involved in the first study. The research group consolidated the planning of the project and received for ethical approval from NSD and Regional Committees for Medical and Health Research Ethics (REK). The first batch of devices was purchased and tested in-lab. The protocol for the first study was defined and reviewed by the user representatives, and information was delivered to the participants accordingly. The recruitment for the first study and obtain oral and written informed consent from the participants is under way.

#### P2 First study

This study (expected N = 15 residents from Helgetun and N = 15 persons with PD) explores the data streams for the devices described in section MATERIALS, to assess their functional and technical validity, and to obtain initial insight on the activity levels of the residents in Helgetun and the symptoms of people with PD. For the latter purpose, the Helgetun residents represent the control group for the participants with PD. Additionally, the development of this first study subsequently informs the practicalities and arrangement of research tasks in the second study (see P5. Development). The first study begins with a baseline data collection that includes a demographic questionnaire to characterise the sample, initial semi-structured questions about their expectations and its technology, and the clinical assessment instruments described previously. Then, the participants simultaneously wear one of each device for 14 days, which passively capture physiological data in their daily life. The researchers visit the participants every 3 days to carry out monitoring tasks: charge the devices, back-up the sensor data, answer questions or resolve issues from the participants, and take notes about their activities since last visit. After the device data collection, we conduct another standardised clinical assessment and a set of in-depth interviews addressing technology adoption, activity levels and current perceived wellbeing of the participants.

The anonymised sensor data is analysed in several steps. First, a correlation analysis compares the data obtained from the different sensors and matches it with the ground truth obtained in the monitoring sessions. Second, we perform a preliminary activity recognition from the sensor data by segmenting, extracting features, and aggregating the different streams (pre-processing). Then, in a cyclic manner, we develop and validate algorithms that detect daytime and night-time activity patterns (rule-based reasoning machine based on moving window Fast Fourier Transform).

The analysis of qualitative data takes place in a thematic and narrative analysis process: open coding, calculating inter-coder agreement, and then axial coding, to finally extract the main categories and narratives. Qualitative data will be interpreted in a hermeneutical approach aiming to explore varieties in the perspectives of the individual study participants and common traits for the group. The process will be cyclic, interpreting each interview as singular texts, the whole body of interviews, each interview in relation to the whole and the whole in relation to each individual interview (53).

#### P3. Transition

This stage includes dissemination of results from P2, purchase of further device batches, application for ethical approval of P4, and recruitment of participants.

#### P4. Second study

We envision 3 data collections at different parts of the year to capture seasonal and intra-subject variances: winter-spring, summer-autumn, and winter-spring again. The arrangement of the data collection tasks will be similar to P2, unless the results of the first study indicate otherwise. Sensor data is collected extensively, aiming to include 90 dyads from the DIGI.PARK branch (person with PD–informal caregiver) and 90 participants from the Helgetun branch (residents and people on the waiting list).

Sensor data analysis begins with the same pre-processing as in P2. In *AI-Driven Activity Recognition* (Fig 4), we will finalize the rule-based reasoning machine from P2 and tune it with machine learning procedures. *Knowledge Discovery* will be performed using classifiers, clustering, and fusion to build digital phenotypes. *Knowledge Correlation Analysis* will be made by matching the digital phenotypes to the qualitative data. *Natural Language Generation* associates dictionary words or idioms to the digital phenotypes and is part of the user interface developed in P5. *Machine Learning* involves designing and optimizing the AI modules that, based on the discovered knowledge, generate machine advice for the participants from the Helgetun branch. For the DIGI.PARK branch, we will build *Inference Prediction Models* based on fuzzy intelligent technology which combines expert knowledge with data-driven knowledge.

#### P5. Development

In parallel to P4, and learning from it, we will undertake a development stage where the digital phenotyping algorithms will be developed and validated in a cyclic process. We plan to develop two main items in this stage: 1) the software that contains and runs the algorithms on the wearable devices and paired mobile phones, as well as a visual interface delivery of lifestyle advice and accessible data from digital phenotypes; 2) the new outcome measures for PD and the algorithms or models that will compute them, as well as an executable version (simple software & interface) to be used in future research. The ActiveAgeing software will be developed following an agile development methodology adapted to mobile eHealth contexts [59]. The new PD outcome measures will be developed using a cyclic data-driven methodology in which algorithms and models are designed, then iteratively adjusted according to testing results and expert feedback, until validated (see Fig 4).

#### P6. Follow-up

This phase contains the results dissemination to popular science and academic targets, user information, and seminars.

## Discussion

The ActiveAgeing study protocol describes our approach that includes older participants with and without PD who live in traditional and innovative living environments, and explores digital phenotyping with wearable devices to deepen the understanding of the ageing process and PD. The following subsections address key practical aspects of this protocol and its approach.

### Practical pitfalls and obstacles

Carrying out digital phenotyping with consumer wearable devices entails practical challenges that must be considered. Using commercial technology means that ergonomics, user-friendliness, or reliability of the devices are not under the control of the researchers. However, this study relies on the idea that using commercial devices is preferable to developing in-house sensors or test custom, non-commercial designs, because this minimises potential stigmatisation and facilitates knowledge and technology transference. Moreover, some of these devices do not provide raw data, but their own calculations from black-boxed algorithms. In our cyclic review, we dropped the device Philips Actiwatch Spectrum Plus, since the movement data and activity count that it provides are calculated in a way that would not allow us to apply DSP to the needed extent. We decided to change it to Empatica E4 before the first study, at the end of the planning stage. Second, digital phenotyping implies collecting large sets of data, which entails a logistic challenge for the second study. Additionally, another pitfall that we must consider is the risk of AI algorithms failing to provide accurate outputs that serve as basis for clinical and lifestyle decision-making. We aim to mitigate this risk by building a cyclic methodology around it that allows us to finetune, re-design and validate our algorithms and models.

### Methodological considerations about AI

The ActiveAgeing study proposes to use AI to enhance clinical assessment made by humans and be less time- and resource-consuming, not to replace the human expertise. Therefore, this study takes a firm stance on the use of explainable AI. Explainable AI (XAI) is a label that describes the subgroup of AI-based techniques whose models and algorithms are not just internal constructs used by the model to produce an output or a classification, but non-opaque, understandable decisions made by the machine [60]. Thus, the human can make an a-posteriori assessment of that machine decision, as well as modify parameters and check biases. Therefore, non-explainable AI models such as support vector machines, neural networks, or deep learning are not prioritised in this study.

### Methodological considerations about the health status of the participants

In studies including older adults and people with neurological diseases such as PD or dementia, age may be a crucial inclusion or exclusion criteria. However, given the timespan (4 years) and the target population of this study (older adults in different stages of their lives), we reconsidered this. Ageing, like life, is not a process that happens only once, and therefore we defend that an initial exclusion based on an assessment of health would not be pragmatic, considering that even initially healthy participants are likely to develop acute or chronic conditions any time during these four years. Thus, changes in health status will not be a reason to exclude a participant, unless it limits life expectancy. The consent can be withdrawn throughout the process and written and oral informed consent will be renewed before every data collection.

### Study status

At the time of writing this paper, the project is in mid-P2 (March 2022), the first study. Ethical approval from NSD has been obtained (RN-792472). The Regional Committee for Medical and health Research Ethics of Vestlandet (REK) deemed not necessary to obtain their approval, since no clinical intervention is applied, and we re-submitted the application with more detail in case the clinical approval was necessary afterwards, but they confirmed their assessment. A total of 17 participants from Helgetun have been recruited, provided written informed consent for the first study, and recruitment for the DIGI.PARK branch has begun.

## Supporting information

S1 Checklist SPIRIT 2013 checklist: Recommended items to address in a clinical trial protocol and related documents*. (DOC)

## Abbreviations

PD: Parkinson’s disease
AI: Artificial Intelligence
DSP: Digital signal processing
GAI: Geriatric Anxiety Inventory
GDS: Geriatric Depression Scale
KDD: Knowledge Discovery from Data
MDS-UPDRS: Movement Disorder Society Unified Parkinson’s Disease Rating Scale
ML: Machine learning
MoCa: Montréal Cognitive Assessment Scale
RBDSQ: REM Sleep Behavior Disorder Screening Questionnaire
SAS: Starkstein Apathy Scale
